# Microbial Production of Melanin Pigments from Caffeic Acid and L-Tyrosine Using *Streptomyces glaucescens* and FCS-ECH-Expressing *Escherichia coli*

**DOI:** 10.3390/ijms22052413

**Published:** 2021-02-27

**Authors:** Soo-Yeon Ahn, Seyoung Jang, Pamidimarri D. V. N. Sudheer, Kwon-Young Choi

**Affiliations:** 1 Environment Research Institute, Ajou University, Suwon 16499, Gyeonggi-do, Korea; sooyeon@ajou.ac.kr; 2 Department of Environmental and Safety Engineering, College of Engineering, Ajou University, Suwon 16499, Gyeonggi-do, Korea; young0845@naver.com; 3 Amity Institute of Biotechnology, AMITY University Chhattisgarh, Raipur 493558, India; pdvnsudheer@gmail.com; 4 Department of Environmental Engineering, College of Engineering, Ajou University, Suwon 16499, Gyeonggi-do, Korea

**Keywords:** eumelanin, allomelanin, melanin-diamine complex, caffeic acid, L-lysine, L-tyrosine

## Abstract

In this study, synthetic allomelanin was prepared from wild-type *Streptomyces glaucescens* and recombinant *Escherichia coli* BL21(DE3) strains. *S. glaucescens* could produce 125.25 ± 6.01 mg/L of melanin with a supply of 5 mM caffeic acid within 144 h. The ABTS radical scavenging capacity of *S. glaucescens* melanin was determined to be approximately 7.89 mg/mL of IC_50_ value, which was comparable to L-tyrosine-based eumelanin. The isolated melanin was used in cotton fabric dyeing, and the effect of copper ions, laccase enzyme treatment, and the dyeing cycle on dyeing performance was investigated. Interestingly, dyeing fastness was greatly improved upon treatment with the laccase enzyme during the cotton dyeing process. Besides, the supply of C5-diamine, which was reported to lead to more complex crosslinking between melanin units, to caffeic acid-based melanin synthesis was also investigated for higher production and novel functionalities. To facilitate the supply of caffeic acid and C5-diamine, *E. coli* strains expressing each or combinations of tyrosine ammonia lyase/*p*-coumarate 3-hydroxylase, feruloyl-CoA synthetase/enoyl-CoA hydratase/aldolase, and tyrosinase/lysine decarboxylase enzymes were prepared and investigated for their eumelanin, C5-diamine, and allomelanin production from L-tyrosine and L-lysine, respectively. Finally, H-NMR, FT-IR, and MALDI-TOF analysis of the synthetic melanin pigments were attempted to obtain the chemical information.

## 1. Introduction

Melanin is one of the most well-known and abundant pigments in nature. The biological function and structure of melanin vary depending on the species, and it generally forms brown and black pigments [1,2,3]. Depending on the type of substrate used for its biosynthesis and synthetic route, melanin pigments can be classified into several groups. In general, dihydroxyphenylalanine (L-DOPA) and L-dopaquinone-based melanin are classified into eumelanin. And allomelanin, which are composed of aromatic monomers with a catechol structure such as caffeic acid and pyomelanin, have also been reported [4,5]. Melanin pigments have a complex structure with varying repeating units that are randomly polymerized. These units include L-DOPA, dopaquinone, dopachrome, and 5,6-dihydroxyinodole which were generated through the tyrosinase dependent L-tyrosine conversion in eumelanin synthesis [1,6]. 

Several melanin-producing strains have been reported. For example, bacteria such as *Streptomyces glaucescens*, *Nocardiopsis alba*, *Pseudomonas stutzeri*, and fungi such as *Armillaria ostoyae*, *Aspergillus funigatus*, and *Daldinia concentrica* have been reported to produce L-DOPA-based eumelanin [7,8,9,10,11,12,13]. Their melanin production titers were reported to range from a few to many g/L, and some showed excellent titers. Not only wild-type based melanin production, but also a recombinant strain expressing enzymes in melanin synthetic pathway were often reported. Recently, the MelC enzyme, a type of tyrosinase derived from Bacillus megaterium, was identified, and was utilized for the biosynthesis of eumelanin in *Escherichia coli* [14,15,16]. Besides, recombinant allomelanin biosynthesis has been reported through generating monomers having a catechol structure derived from caffeic acid and protocatechualdehyde [17]. Recently, Jang et al. reported the allomelanin production with 0.2 g/L of titer by constructing feruloyl-CoA synthetase (FCS) and enoyl-CoA hydratase/aldolase (ECH) expression system in *E. coli* which could bioconvert caffeic acid into allomleanin repeating unit of protocatechualdehyde [18]. Also, heterologous expression of 4-hydroxyphenyl pyruvate dioxygenase (HPPD) in *E. coli* to synthesize homogentisate from L-tyrosine was reported to result in 0.315 g/L of pyomelanin production [19]. 

Along with melanin production using microbial, application of melanin pigments has been greatly attempted. Melanin extracts from natural and synthetic sources, for example, have been utilized as UV-protective films and coating materials owing to their excellent physical and biological functionalities. Recently, an attempt to design and engineer the physiological functionalities of melanin by introducing new radical precursors into melanin synthetic pathways has been reported. For example, 3-hydroxyindole was introduced by expressing a cytochrome P450 monooxygenase (CYP) enzyme that mediates a site-specific hydroxylation reaction to indole C3 position in eumelanin biosynthesis, and the generated novel melanin showed different physical functionalities [20]. Also, electrochemical properties of synthetic melanin have been reported to widen the sphere of its application to the field of electrochemistry [21,22,23]. Besides, it could also be used for pharmaceutical and cosmetic materials owing to its high biocompatibility [24,25,26].

Such an attempt to engineer the functionality of synthetic melanin, in this study, novel melanin pigments were synthesized and applied for fabric dyeing. First, wild-type *S. glaucescens* melanin was investigated for melanin production. Cells were cultured in a medium containing caffeic acid to biotransform caffeic acid into allomelanin. The generated melanin was isolated and used for cotton fabric dyeing. After washing the melanin-dyed fibers, the dyeing fastness was determined through a color difference test. The fastness was investigated against several dyeing conditions such as copper ions addition, multi-dyeing cycles, and laccase enzyme treatment. 

Next, recombinant strains were constructed to establish a system capable of simultaneously producing eumelanin and allomelanin in *E. coli* host cells and to engineer the structural properties of repeating units. To facilitate this, a system capable of supplying C5-diamine (cadaverine) or generating it from L-lysine was attempted to introduce cross-link reagent in melanin synthesis. Eumelanin was supplied from L-tyrosine through the MelC reaction, and protocatechualdehyde-based allomelanin was supplied from L-tyrosine through the simultaneous enzymatic reaction of tyrosine ammonia-lyase (TAL) and *p*-coumarate 3-hydroxylase (C3H). Also, the crosslinking reagent of C5-diamine was supplied from L-lysine through the enzymatic reaction of lysine decarboxylase (CadA) [27]. A system capable of producing melanin-diamine complexes in a single whole cell reaction was constructed investigated for its melanin production. Finally, structural understandings of the synthesized melanin pigments were attempted by H-NMR and FT-IR analysis. The results of this study were expected to contribute to future research on functional polymers and fibers containing microbial melanin and the development of a platform strain capable of synthesizing new functional melanin.

## 2. Results

### 2.1. S. glaucescens Culture and Caffeic Acid-Based Melanin Production 

**Figure 1:**
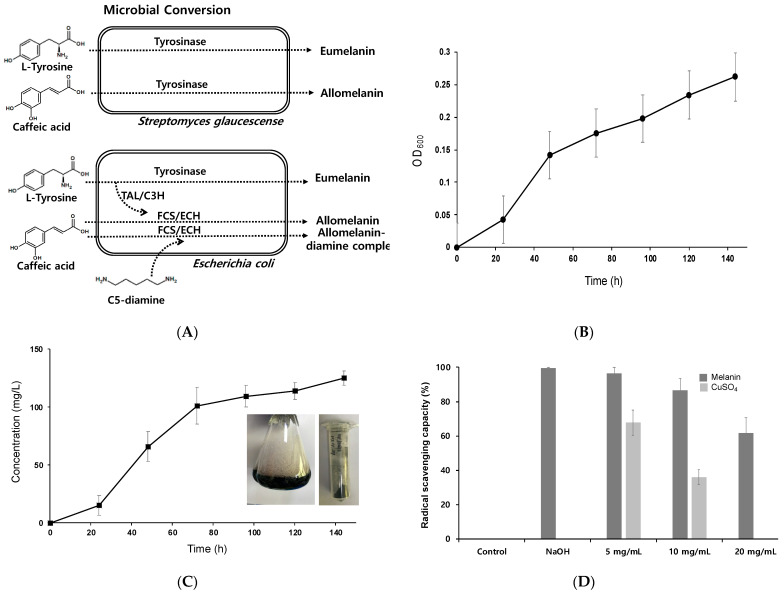
(**A**) Microbial conversion of L-tyrosine and caffeic acid by *Streptomyces glaucescens* and engineered *E. coli*. To prepare the microbial melanin pigments, 5 mM of caffeic acid was supplied to the culture medium and the isolated melanin pigment was dried for further experiments. (**B**) Time-dependent growth curve of *S. glaucescens*. (**C**) Time-dependent production of caffeic acid-based melanin. The highest melanin production titer was achieved at 125.25 ± 6.01 mg/L at 144 h of incubation. (**D**) Determination of radical scavenging capacity of melanin.

Previously bioproduction of melanin using *S. glaucescens* from protease peptone was reported to result in 350 mg dry wt/L of titer [7]. This suggested microbes could be used as a conversion toolbox for melanin pigment production. To facilitate this, wild-type *S. glaucescens* and recombinant *E. coli* strains were investigated for caffeic acid-based melanin synthesis with the supplementation of C5-diamine (Figure 1A). First, caffeic acid-based allomelanin was produced through *S. glaucescens* whole-cell biotransformation. *S. glaucescens* was cultured at 30 °C for 5 days, and 5 mM caffeic acid was added to the culture solution for biotransformation. Cells grew to an OD_600_ of 0.26 for 142 h and a dry weight of 0.17 mg/L could be obtained (Figure 1B). To measure the amount of melanin produced, 1 mL of each sample was collected every 24 h and quantitatively analyzed. Melanin production increased rapidly between 24 h and 72 h during biotransformation, and the highest titer of 125.25 ± 6.01 mg/L could be achieved at 144 h (Figure 1C). Based on the caffeic acid fed to the medium, the final yield was calculated as 0.14 g melanin/g caffeic acid.

### 2.2. ABTS Radical Scavenging Capacity of Caffeic Acid-Based Melanin 

Melanin pigment has been shown to have antioxidant properties [7,28,29,30,31]. In particular, eumelanin synthesized through L-tyrosine biotransformation in *E. coli* has been reported to exhibit antioxidant efficacy at an IC_50_ level of 200–300 μg/mL, while homogentisate-based pyomelanin from L-tyrosine had an IC_50_ level of approximately 100 μg/mL [7,17]. In this study, the ABTS radical removal capacity of *S. glaucescens* melanin was determined as an IC_50_ value of 25.08 mg/mL and 7.89 mg/mL in the presence of copper ions (Figure 1D). These were 10 and 4 times higher than those of eumelanin and pyomelanin in the absence of copper ions, respectively.

### 2.3. Dyeing Performance and Fastness of Synthetic Melanin on Cotton 

**Figure 2:**
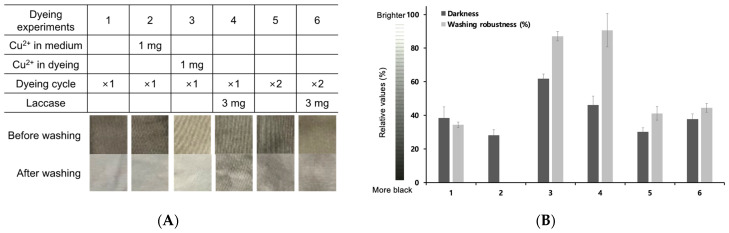
(**A**) Dyeing of *S. glaucescens* melanin on cotton fabric at different conditions: (1) dyeing with the culture supernatant without copper ions, (2) dyeing with the culture supernatant where melanin was synthesized by adding copper ions to the culture solution, (3) copper ions added to the melanin supernatant during dyeing process, (4) 3 mg (1.5 U) of laccase enzyme treatment to the melanin supernatant during dyeing, (5) double dyeing cycles without copper and laccase enzyme treatment and (6) 3 mg (1.5 U) of laccase enzyme treatment during dyeing and the double dyeing cycles. (**B**) Dyeing performances and dyeing fastness of caffeic acid-based melanin on cotton fabric. The dyeing performance was measured based on the brightness of L in Table 1, and the dyeing fastness was determined through the equation: 100 + [(L + L’)/L] × 100(%), where L is the brightness before washing and L’ is the brightness after washing.

*S. glaucescens* melanin was applied for cotton dyeing and use as functional fibers. The manufacture of functional cotton materials dyed with melanin was conducted based on the results of the evaluation of the cotton fiber dyeing performance of previously reported indigo-based dyes [20]. A 10 cm × 10 cm cotton sheet was prepared and stained with the melanin culture supernatant first. The dyeing was carried out at different reaction conditions with heating at 50 °C for 2 h (Figure 2A). The dyeing performance of cotton, which was visually checked immediately after dyeing and drying, was found to be the lowest in cotton 3, in which copper ions were added during dyeing. To quantitatively evaluate the dyeing performance, color differences with specific values were determined (Table 1). In particular, the L value representing the brightness was quantitatively compared for each of the stained cotton sheets (1–6), and a darker color was displayed numerically. The addition of copper ions to the cotton without any treatment in the melanin supernatant during the dyeing process showed the lowest L value (#2 in Figure 2A), suggesting the highest dyeing performance.

Next, each of the dyed cotton samples was washed with distilled water to measure the dyeing fastness before and after washing (Figure 2B, bright bars). Fastness was reported as a percentage by quantifying the L value before and after washing according to the equation: 100 + [(L − L’)/L] × 100(%), (L; before washing, L’; after washing). Not only did the L value show a distinct distribution depending on the dyeing process, but the fastness also showed a deviation of up to 80% or less than 40%. Interestingly, laccase enzyme treatment during the dyeing process greatly increased the robustness of cotton 4 (90.6%). Laccase enzyme treatment might induce radical formation in the melanin structure, leading to the tighter binding of melanin to the cellulose constituting the cotton fiber tissue. According to previous reports of laccase-mediated oxidation of DOPA for coloration of silk fabric, the addition of laccase could catalyze the oxidation of melanin and enhance its polymerization, followed by coloration [32,33,34]. When copper ions were added during dyeing, the color fastness was 86.9%. In other cases, there was no significant difference in fastness. However, when copper ions were added during culture, both dyeing performance and fastness were low.

### 2.4. Caffeic Acid Melanin Complex Production 

**Figure 3:**
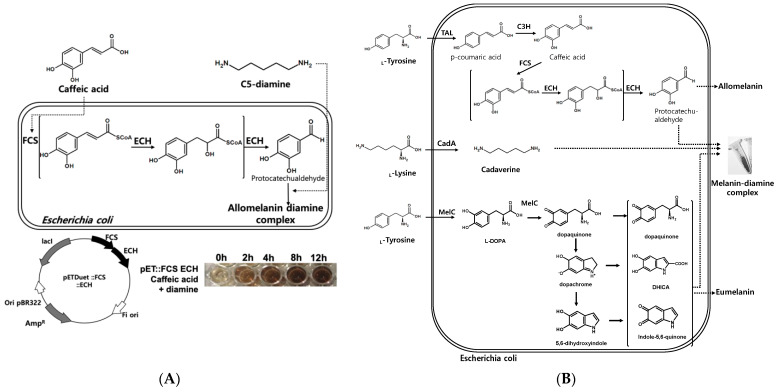
(**A**) Biosynthesis of the allomelanine-C5 diamine complex using *E. coli* cells expressing FCS and ECH enzymes, and production of the allomelanin-diamine complex from 5 mM caffeic acid and diamine. (**B**) Co-production of the eumelanin-allomelanin-diamine complex by feeding L-tyrosine and L-lysine into *E. coli* cells expressing tyrosinase (MelC), tyrosine ammonia-lyase (TAL), C3H, FCS, ECH, and CadA simultaneously, respectively.

According to a recently published paper, the addition of C6-diamine (hexamethylenediamine) to caffeic acid can induce cross-linking to form a film [35]. Based on previous results where caffeic acid-based allomelanin was produced using wild type *S. glaucescens*, engineered allomelanin biosynthesis in a recombinant *E. coli* system was attempted. Two enzymatic reactions (FCS and ECH) were introduced in an *E. coli* host to convert the carboxylic acid functional groups into aldehyde as the aldehyde functional group can undergo reductive amination with diamine cross-linkers, which could form allomelanin-diamine complexes (Figure 3A). The host cells expressing genes encoding FCS and ECH were incubated with a co-supply of caffeic acid and C5-diamine. The cells could produce allomelanin-diamine complexes up to 0.251 g/L in 12 h (Figure 3B). This was a 25% increase compared to the production of 0.2 g/L of allomelanin in the absence of diamine [18]. Based on the substrate fed to the medium, a final yield of 0.178 g of melanin/g substrate could be obtained.

### 2.5. Construction of a Synthetic Pathway for the Formation of the Melanin-Diamine Complex from L-Tyrosine and L-Lysine

As caffeic acid and C5-diamine are platform chemicals that can be used in a variety of industries and can provide high added value, it is possible to supply L-tyrosine, and L-lysine amino acids as precursors instead. First, L-tyrosine was converted to caffeic acid through TAL and C3H enzyme reaction, and L-lysine to C5-diamine through the CadA (lysine decarboxylase)-dependent decarboxylation reaction, respectively. Therefore, it was possible to produce caffeic acid-based allomelanin from L-tyrosine and L-lysine through a combination of TAL, C3H, FCS, ECH, and CadA enzyme reactions (Figure 3B).

Generally, eumelanin is an L-tyrosine-based pigment, where tyrosinase-dependent oxidation is responsible for its synthesis. The tyrosinase enzyme catalyzes the oxidation of L-tyrosine converting it into L-DOPA in an ortho-specific manner, followed by sequential oxidation of L-DOPA into dopaquinone which is further oxidized into dopaquinone radicals. These L-tyrosine-derived intermediates, dopaquinone, 5,6-dihydroxyindolecarboxylic acid (DHICA), and indole-5,6-quinone derived radicals, are polymerized randomly by each other, resulting in the formation of eumelanin polymer. This suggested that eumelanin could be produced simultaneously with allomelanin by expressing the tyrosinase MelC enzyme in the presence of L-tyrosine. Through the construction of the biosynthetic pathways of eumelanin and allomelanin together, biosynthesis of a novel melanin complex could be achieved through L-tyrosine and L-lysine co-biotransformation.

**Figure 4:**
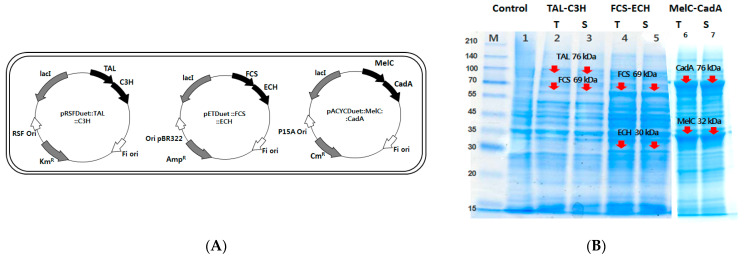
(**A**) Construction of TAL-C3H, FCS-ECH, and MelC-CadA co-expression plasmids. (**B**) SDS-PAGE analysis of TAL, C3H, FCS, ECH, MelC, and CadA enzyme co-expression in *E. coli* BL21(DE3). Control indicates *E. coli* BL21(DE3) cells without protein overexpression. Each T and S indicates the total and soluble fraction in each protein, respectively.

To co-produce the allomelanin, eumelanin, and diamine complex simultaneously in a single *E. coli* strain, each gene expressing TAL and C3H, FCS and ECH, and MelC and CadA were cloned in the pRSFDuet, pETDuet, and pACYCDuet vectors, respectively (Figure 4A). The expression of each of the six proteins in a single melanin-producing host strain was verified by SDS-PAGE analysis. It was confirmed that each protein with a molecular weight of TAL (76 kDa), FCS (69 kDa), ECH (30 kDa), MelC (32 kDa), and CadA (76 kDa) was well expressed as a soluble fraction and found at the corresponding molecular weight at SDS-PAGE (Figure 4B).

### 2.6. Effect of TAL/C3H, FCS/ECH, and CadA Enzymes on the Production of the Melanin-Diamine Complex

**Figure 5:**
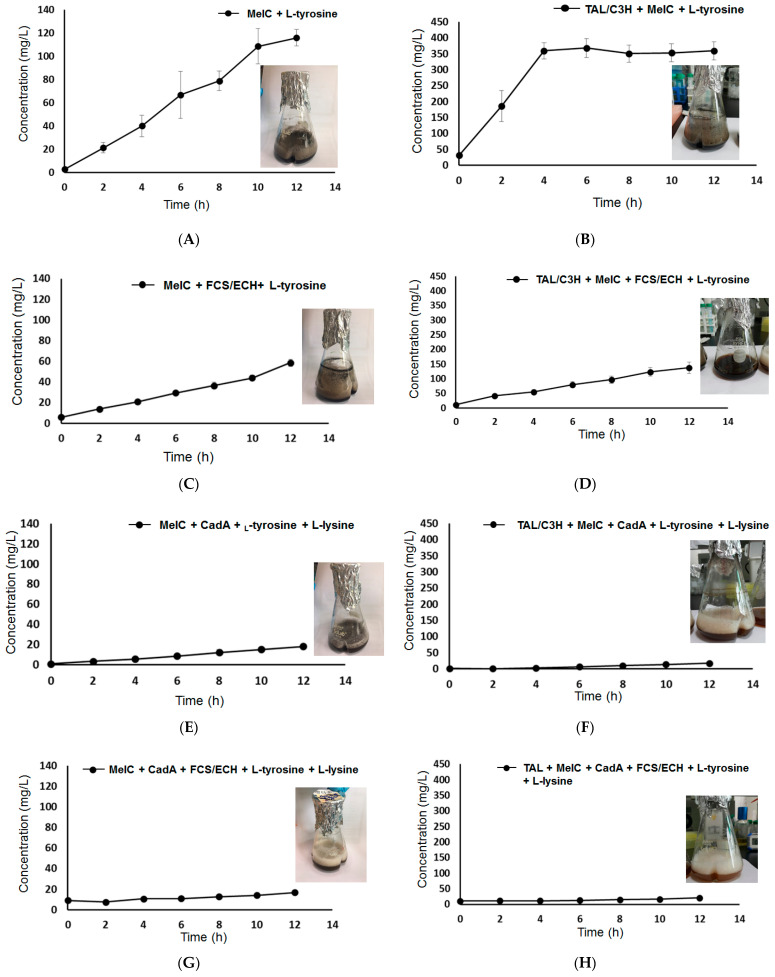
Eumelanin and melanin-diamine complex production profile in various enzyme expression levels. The detailed information of overexpressed enzymes in figure (**A**–**H**) and approximate titers are summarized in Table 2.

Quantitative analysis was performed on the generation of melanin-diamine complexes depending on the expression of TAL/C3H, FCS/ECH, and CadA enzymes in the melanin-producing *E. coli* host. When MelC was solely expressed with 5 mM L-tyrosine, approximately 120 mg/L of melanin was produced (Figure 5A, Table 2 #5). It seemed that only eumelanin was produced because allomelanin and diamine-producing enzymes and substrates were not supplied. On the other hand, when MelC and TAL/C3H were simultaneously expressed, approximately 370 mg/L of melanin was produced (Figure 5B, Table 2 #1). In this case, diamine was not supplied, but since the catechol of caffeic acid was supplied from L-tyrosine through the TAL/C3H enzyme reaction, it might have a positive effect on the production of the melanin complex.

When 5 mM tyrosine was supplied as a substrate while simultaneously expressing TAL/C3H, MelC, and FCS/ECH, the amount of melanin was reduced to 140 mg/L compared to when the FCS/ECH enzyme was not expressed (Figure 5D, Table 2 #2). The melanin complex was produced from the supply of eumelanin and protocatechualdehyde generated through the TAL/C3H-FCS/ECH enzyme reaction, but it might also be due to the low conversion activity resulting from the decrease in the amount of enzyme expression. In particular, when only MelC and FCS/ECH, excluding TAL/C3H, were expressed in the same reaction, the production of the melanin complex decreased dramatically to less than 60 mg/L (Figure 5C, Table 1 #6). It could be possible to synthesize eumelanin from L-tyrosine via MelC expression; however, due to the simultaneous expression of the FCS/ECH enzyme, the level of MelC enzyme expression decreased and the conversion activity decreased accordingly. This could be due to unknown side reactions with intracellular metabolites.

In the case of allomelanin, diamine, and eumelanin co-production, all pathways from L-tyrosine, L-lysine, the six enzymes TAL/C3H, FCS/ECH, CadA, and MelC were expressed. When 5 mM of L-tyrosine and L-lysine was supplied, the amount of melanin complex produced was approximately 20 mg/L, or less (Figure 5H, Table 1 #4). This was a significantly lower production level compared to that obtained with the expression of MelC alone or that of MelC, TAL/C3H, and FCS/ECH without diamine supply. This could be because the expression level of melanin was remarkably reduced when the six enzymes were simultaneously expressed in a single host, or that the enzymatic conversion was remarkably decreased. In addition, the melanin complex production was also at a significantly low level (below 20 mg/L) in strains concurrently expressing MelC and CadA (Figure 5E, Table 1 #8), TAL/C3H (Figure 5F, Table 1 #3), and FCS/ECH (Figure 5G, Table 1 #7) enzymes.

Overall, simultaneous L-lysine supply and CadA expression led to low melanin complex production, at 20 mg/L or less. In addition to the low conversion activity, cytotoxicity due to C5-diamine formed by the CadA enzyme was also recorded. Recent studies have shown that *E. coli* resistance to C5-diamine is quite low. Exposure to 0.3–0.5 mol/L C5-diamine for 8 h has been reported to induce rapid cell growth inhibition and cell disruption [36]. Therefore, further studies on intracellular diamine concentration control acting as a cross-linker for effective diamine supply and melanin complex formation are needed.

### 2.7. Structural Analysis of Melanin Pigments

**Figure 6:**
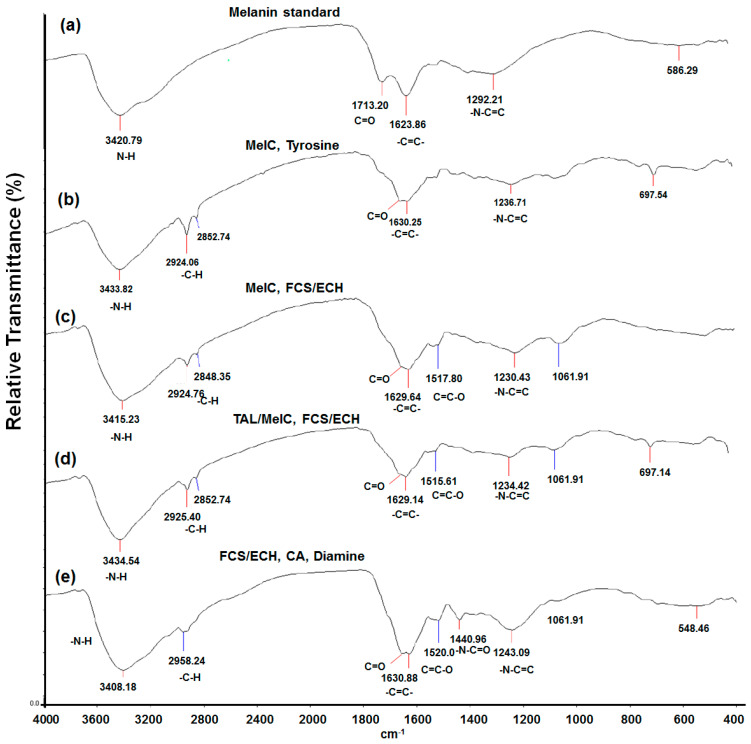
IR spectra of (**a**) commercial melanin as a control, (**b**) MelC, tyrosine, (**c**) MelC, FCS/ECH, and tyrosine, (**d**) TAL/MelC, FCS/ECH, and (**e**) FCS/ECH, caffeic acid, and diamine.

To investigate the chemical structure of the synthesized melanin pigments ^1^H NMR and FT-IR analysis was conducted first. ^1^H-NMR analysis resulted in similar patterns of chemical shifts between melanin standard as a control and synthetic melanin pigments (Figure S1). The melanin standard was commercial and prepared by tyrosine oxidation by hydrogen peroxide. In results, both showed obvious chemical shifts around 7.0–7.1 ppm originated from Ph-OH of benzaldehyde and indole repeating unit. Also, several minor shifts were observed at aliphatic regions (0.8–1.3 ppm), which are not sufficient to interpret the chemical shift to structural correlation. On the other hand, FT-IR analysis showed characteristic wavenumber of 2850, and 2920 cm^−1^ in synthetic melanin pigments which were not observed in melanin standard and representative of sp^2^ C-H stretch (Figure 6b–e). Instead, melanin standard showed wavenumber found in all melanin samples at 3420, 1713, 1623, and 1292 cm^−1^, which represented N-H, C=O, C=C, and C-N stretch, respectively (Figure 6a). Interestingly, melanin synthesized by FCH/ECH expressing cells showed distinctive wavenumber at 1515–1520 cm^−1,^ and melanin with additional diamine showed another one at 1440.9 cm^−1^, which represents O-C=C and N-C=C bonding, respectively (Figure 6c,d). Moreover, a unique wavenumber at 1440 cm^−1^ was observed only in diamine-containing melanin, which represented –N-C=O amide bonding (Figure 6e).

According to the previous report of caffeic acid esters with diamines, several representative components of MC/HMDA polymer were proposed, however, not the exact chemical structure was not fully elucidated yet [35]. Besides, several structural analyses by FT-ICR (Fourier-transform ion cyclotron resonance) mass spectrometry were performed in our previous study, however not enough information on the synthetic melanins could be obtained due to the complexity and difficulties in the analysis [17]. To achieve perfect understandings of melanin structure and structure-functionality correlation, further in-depth investigation would be necessary. 

## 3. Discussion

In this study, caffeic acid-based synthetic melanin pigments were synthesized using wild-type *S. glaucescens* and recombinant *E. coli* strains from caffeic acid or the amino acids L-tyrosine and L-lysine. The synthesized melanin by *S. glaucescens* was further investigated for its functionalities, such as ABTS radical scavenging capacity and dyeing performance. Interestingly, an additional supply of laccase enzyme into dyeing performance positively affected the dyeing performances by leading to the oxidation of melanin and polymerization. 

However, the use of *S. glaucescens* for melanin production, next recombinant *E. coli* strains with additional linker supplement were utilized and investigated for their melanin production. Besides, the supply of C5-diamine, which was reported to lead to more complex crosslinking between melanin units, to caffeic acid-based melanin synthesis was also investigated for higher production and novel functionalities. First, the supply of C5-diamine increased the melanin production titer in the recombinant *E. coli* system expressing FCS and ECH enzymes. Next, for the further development of diamine, eumelanin, and allomelanin complex synthesis, expression of several combinations of enzymes responsible for melanin monomer and linker supply were investigated, followed by quantifying the melanin produced. Based on the results of this study, the potential to use melanin pigments synthesized from microorganisms as functional dyeing materials was confirmed. In terms of titer, the TAL/C3H and MelC combination, with a supply of L-tyrosine showed the highest melanin production of approximately 400 mg/L.

In recent years, environmental pollution and ecosystem destruction due to the discharge of textile dyes to aquatic ecosystems have been observed. From this point of view, it is expected that fastness can be improved through laccase enzyme treatment during the dyeing process and that dyeing efficiency can be improved through the introduction of a continuous dyeing cycle to develop an eco-friendly process throughout the dyeing cycle. Besides, melanin is a promising biomaterial that could be used for different purposes. It would be possible to use melanin-diamine complexes as functional films and coating materials, after the characterization of the physical properties of melanin-diamine composite polymers in the future. Although its chemical structure and structure-functionalities are not yet fully understood, engineering studies to obtain desirable production titers and novel functionalities of biomaterial melanin complexes would be very promising further studies.

## 4. Materials and Methods

### 4.1. Strains and Chemicals

The *Streptomyces glaucescens* KCTC988 strain used in the experiment was purchased from the Center for Biological Resources (KCTC, Korean Collection for Type Cultures). *E. coli* DH5α and *E. coli* BL21 (DE3) were used to construct plasmids. *Burkholderia glumae* BGR1 strain was purchased from the Korean Collection for Type Cultures (KCTC). L-tyrosine, L-lysine, caffeic acid, diaminopentane (cadaverine), isopropyl *β*-D-1-thiogalactopyranoside (IPTG) used for melanin synthesis, and Melanin standard (Cas. 8049-97-6) were all purchased from Sigma-Aldrich Korea (Suwon, Gyeonggido, Korea).

### 4.2. Construction of Expression Plasmids of TAL-C3H, FCS-ECH, and MelC-CadA Coding Genes

Genes encoding TAL and C3H enzymes derived from *Rhodotorula glutinis* and *Saccharothrix espanaensis* strains were synthesized by optimizing them based on codon usage (Bioneer, Daejeon, Korea). The TAL and C3H coding genes amplified via PCR were cloned into the multi-cloning site (MCS) of the pRSFduet-1 vector. The genes encoding FCS and ECH enzymes were derived from the *B. glumae* BGR1 strain, and the genes obtained through PCR from the *B. glumae* BGR1 genome were cloned into the MCS of the pETDuet-1 vector, respectively [18]. Also, the genes encoding MelC and CadA were derived from *Bacillus megaterium* and *Klebsiella pneumoniae* strains, respectively, and the genes amplified by PCR were cloned into the MCS of the pACYCDuet-1 vector [37]. 

### 4.3. Cell Culture, Protein Expression, and SDS-PAGE Analysis

The culture medium for *S. glaucescens* was composed of glycerol 15 g/L, L-asparagine 0.5 g/L, K_2_HPO_4_ 0.5 g/L, MgSO_4_·7H_2_O 0.5 g/L, and FeSO_4_·7H_2_O 0.01 g/L [7]. For melanin production, 5 mM caffeic acid was added to the culture medium. After 2 mL of the initial culture solution was incubated at 30 °C for 2–3 days, 0.1% (*v*/*v*) was inoculated into 50 mL fresh culture medium and incubated at 30 °C. Cultures were incubated while checking the cell growth and melanin production up to 144 h after inoculation. A 1-mL sample of the culture was collected every 24 h and used for the quantitative analysis of melanin. 

*E. coli* BL21(DE3) harboring both pETDuet::*fcs*::*ech*-pRSFDuet::*tal*::*c3h* and pACYCDuet::*melC*::*cadA* plasmids were cultured as follows. After incubating 2 mL of the initial Luria-Bertani (LB) medium at 37 °C for 24 h, 0.1% (*v*/*v*) was inoculated into 50 mL of fresh LB medium and incubated at 37 °C. When the OD_600_ reached 0.8, 0.1 mM IPTG was injected into the culture medium so the expression of the protein regulated by the T7 promoter was induced. Then, the incubation temperature was lowered to 30 °C and the cultures were incubated for an additional 12 h. Next, SDS-PAGE analysis was performed to confirm protein expression under the different growth conditions. After incubation, the cultures were centrifuged for 10 min at 13,200 rpm. The supernatant was discarded, and the cells were washed twice with 20 mL of phosphate-buffered saline (PBS) and resuspended in 10 mL sonication buffer. For SDS-PAGE analysis, 1.5 mL of the cell solution was sonicated to disrupt the cells, and the total protein fraction and the soluble fraction obtained after centrifugation at 13,200 rpm for 20 min were used for SDS-PAGE analysis. At this time, wild-type *E. coli* BL21 (DE3) cells were subjected to the same procedure to obtain the total and soluble fractions and used as the SDS-PAGE control.

### 4.4. Bioproduction of Melanin through the Whole Cell Biotransformation of Substrates

*E. coli* BL21(DE3) strains containing or all of pETDuet::*fcs*::*ech*, pRSFDuet::*tal*::*c3h*, pACYCDuet::*melC*::i plasmid, expressed proteins according to the aforementioned method. For the whole cell reaction, caffeic acid, L-tyrosine, and L-lysine were injected into the reaction solution to a final concentration of 5 mM, after resuspension in 10 mL M9 minimal medium. The whole cell reaction was further cultured for at least 12 h more while confirming the melanin production of each culture sample incubating with shaking at 200 rpm at 30 °C.

### 4.5. Extraction and Quantification of Melanin

After the addition of 5 mM caffeic acid, the samples for each period were centrifuged at 5000× *g* for 15 min to separate the supernatant. HCl (6 M) was added to the supernatant to adjust the pH to 2 and then allowed to stand at room temperature for 4 h to precipitate. After precipitation of the melanin sample, the precipitated melanin was separated via centrifugation at 9000× *g* for 15 min. The extracted melanin was washed three times with purified water, centrifuged, and dried at 60 °C for 24 h to obtain granular melanin. Melanin was quantitatively analyzed by measuring the weight of melanin obtained in the dry state. 

During the whole-cell reaction, samples at each time point were centrifuged at 5000× *g* for 15 min, and the supernatants were treated with 6 M HCl to lower the pH to 2 and precipitate melanin. After precipitation of the melanin sample for 4 h, the precipitated melanin was separated via centrifugation at 9000× *g* for 15 min [17,18]. The extracted melanin was washed three times with purified water and dried at 60 °C for 24 h. The produced melanin was quantitatively analyzed by measuring the absorbance at an optical density (OD) of 400 nm. After the whole cell reaction, the cells were separated via centrifugation at 13,200 rpm for 10 min, and the supernatant was used for the quantitative analysis of melanin. Melanin was quantified by applying a conversion constant of 0.066 g/L per unit of OD_400_ [17,18]. The characteristic and structural features of synthetic melanins were analyzed by ^1^H NMR and Fourier-transformed Infrared (FT-IR) analysis.

### 4.6. Determination of the ABTS Radical Scavenging Capacity of Melanin

To evaluate the radical removal performance of melanin, dried melanin was dissolved in 5% NaOH and prepared as a 1 g/L solution. The reaction solution was prepared by mixing an ABTS solution diluted at a concentration of 8.12 mg/mL in phosphate-buffered saline (PBS) with an aqueous solution of K_2_S_2_O_8_ at a concentration of 1.32 mg/mL at 1:1 [17,18]. After mixing, the pH was adjusted to 7.4, and the reaction was started by adding melanin at concentrations of 5, 10, and 20 mg/L to the reaction solution. The reaction was carried out at 25 °C for 24 h while blocking sunlight. After completion of the reaction, the aqueous reaction solution was diluted by adjusting the absorbance to 0.7, and the absorbance was measured at 734 nm to determine ABTS radical scavenging capacity through the equation [(B-S)/B] × 100(%), where B is the absorbance of ABTS and S is the absorbance of the sample + ABTS [38]. 

### 4.7. Fabric Dyeing Using Melanin 

The melanin obtained in a dry state was subjected to a dyeing performance test on cotton fibers. When dyeing directly using the culture medium, cotton fibers were added to 50 mL of the culture supernatant separated through centrifugation, and the reaction was performed at 50 °C for 2 h. An aqueous melanin solution at a concentration of 125 mg/L was used for dyeing. To confirm the effect of copper ions during dyeing, 1 mg CuSO_4_ was added before the start of the reaction. To evaluate the staining effect after the addition of the laccase enzyme, 1.5 U of the laccase enzyme (0.5 U/mg) derived from *Trametes versicolor* was added to the melanin solution, followed by staining. After dyeing, the cotton fibers were dried at 60 °C for 24 h.

### 4.8. Color Difference Test and Determination of Dyeing Fastness to Evaluate Dyeing Performance

To evaluate the dyeing performance, the fibers were washed two to three times with purified water and then dried in the same manner. For efficient dyeing, two cycles of dyeing, drying, and washing were performed using the melanin supernatant. The color difference of the cotton after dyeing was measured using a Colorimeter JZ-600 instrument (Shenzhen Kingwell Instrument Co., Ltd., Guangdong, China) and it’s Color Analysis Management Software [17]. Also, the fastness using the color difference before and after dyeing was calculated as 100 + [(L + L’)/L] × 100(%), where L is the brightness before washing, L’ is the brightness determined after washing, and the fastness of melanin staining was calculated based on 100% when there was no color difference.

## Figures and Tables

**Table 1:** Color differences of *S. glaucescens* melanin-dyed cotton samples.

Cotton Samples		L^1^	a^2^	b^3^	∆E	Washing Robustness (%)
	1	38.45 ± 6.64	0.97 ± 0.43	6.96 ± 0.93	39.08 ± 6.69	ND^4^
	2	28.12 ± 3.37	0.31 ± 0.23	6.93 ± 0.53	28.96 ± 3.39	ND
Before	3	61.77 ± 2.73	−0.75 ± 0.28	14.67 ± 0.25	63.50 ± 2.60	ND
washing	4	46.13 ± 5.37	−0.83 ± 0.63	10.19 ± 0.44	47.26 ± 5.16	ND
	5	30.15 ± 2.45	−1.02 ±0.05	8.06 ± 0.02	31.23 ± 2.36	ND
	6	37.71 ±3.15	0.54 ± 0.02	11.59 ± 0.06	39.46 ± 2.99	ND
	1	63.71 ±1.74	0.72 ± 0.81	3.34 ± 0.19	63.80 ± 1.74	34.3
	2	67.05 ± 0.26	−1.12 ± 0.04	4.05 ± 0.11	67.18 ± 0.25	0
After	3	69.81 ± 2.79	−0.94 ± 0.23	8.19 ± 0.22	70.29 ± 2.75	87.0
washing	4	50.44 ± 9.87	−0.96 ± 0.04	8.71 ± 0.18	51.21 ± 9.69	90.7
	5	47.90 ±4.06	0.17 ± 0.35	6.67 ± 0.03	48.36 ± 4.02	41.1
	6	58.64 ±2.57	−0.02 ± 0.36	7.00 ±0.04	59.05 ± 2.55	44.5

L^1^: brightness, a^2^: the closer to +, the redder, the closer to −, the more green; b^3^: the closer to +, the more yellow, the closer to −, the bluer. ΔE*_ab_ = [(ΔL*)^2^ + (Δa*)^2^ + (Δb*)^2^]^½.^ ND^4^: not determined.

**Table 2:** Production of melanin complexes by *E. coli* depending on the MelC, TAL/C3H, CadA, and FCS/ECH enzyme modules from L-tyrosine and L-lysine.

#	Overexpressed Enzymes	Melanin Unit	Substrate	Production (mg/L)
1	TAL/C3H, MelC	Caffeic acid	L-tyrosine	<370
2	TAL/C3H, MelC, FCS, ECH	Protocatechualdehyde	L-tyrosine	<140
3	TAL/C3H, MelC, CadA	Caffeic acid	L-tyrosine, L-lysine	<20
4	TAL/C3H, MelC, CadA, FCS/ECH	Protocatechualdehyde	L-tyrosine, L-lysine	<20
5	MelC	L-DOPA	L-tyrosine	<120
6	MelC, FCS, ECH	L-DOPA	L-tyrosine	<60
7	MelC, CadA, FCS, ECH	L-DOPA	L-tyrosine, L-lysine	<20
8	MelC, CadA	L-DOPA	L-tyrosine, L-lysine	<20

## Data Availability

Data supporting reported results will be provided upon request.

## Supplementary Materials

The following are available online at https://www.mdpi.com/1422-0067/22/5/2413/s1.
