# Supplementary material for: Microbial Production of Melanin Pigments from Caffeic Acid and L-Tyrosine Using Streptomyces glaucescens and FCS-ECH-Expressing Escherichia coli
Source: Int J Mol Sci. 2021 Feb 27;22(5):2413. doi: 10.3390/ijms22052413 (PMC7957706; doi:10.3390/ijms22052413)
Supplement: Supplementary file 1 [file ijms-22-02413-s001.pdf]

**Figure S1.** $^{1}$H-NMR of (A) melanin standard as a control, (B) MelC, FCS/ECH+tyrosine, (C) TAL/MelC, FCS/ECH

**(A)**

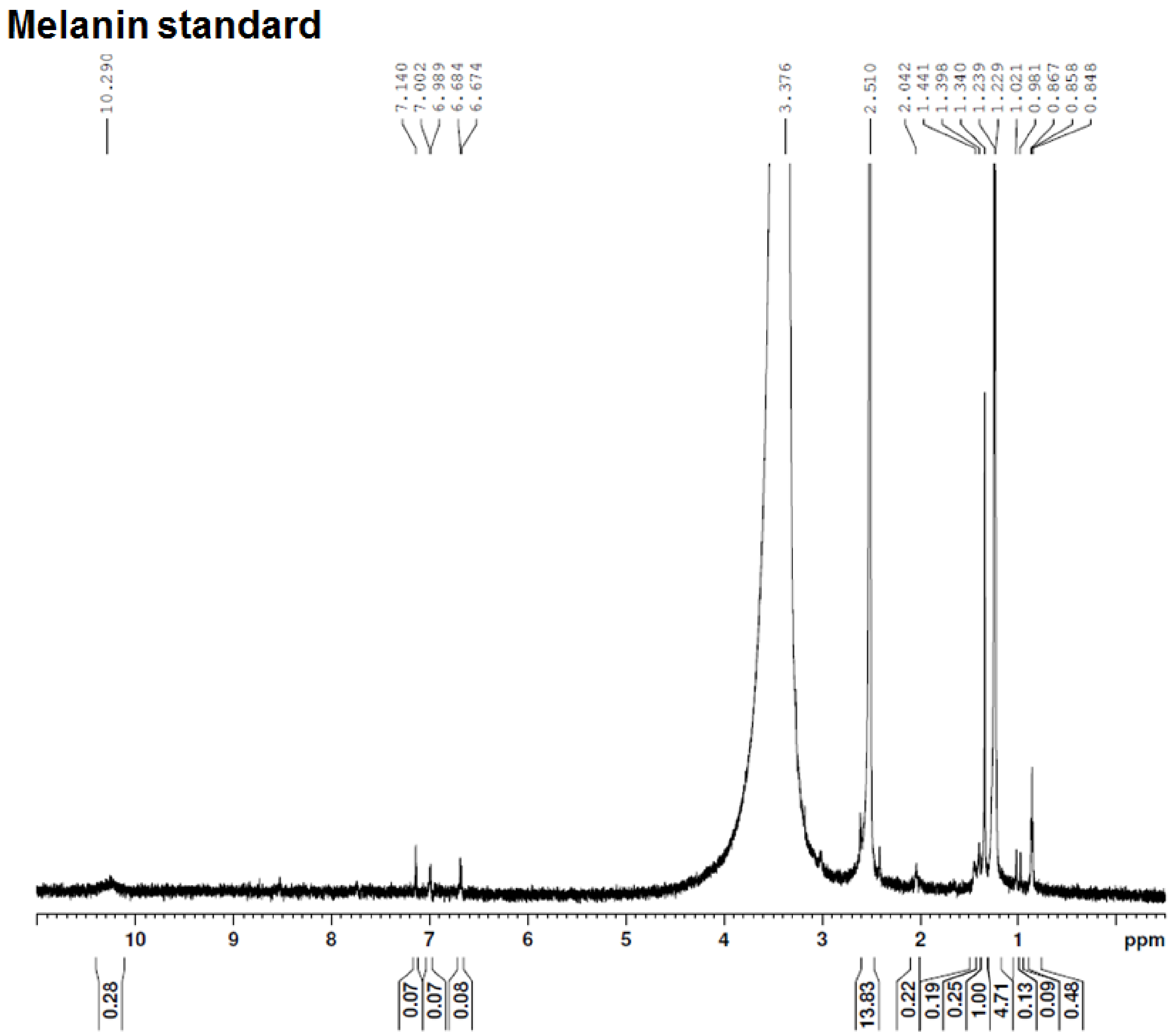

**(B)**

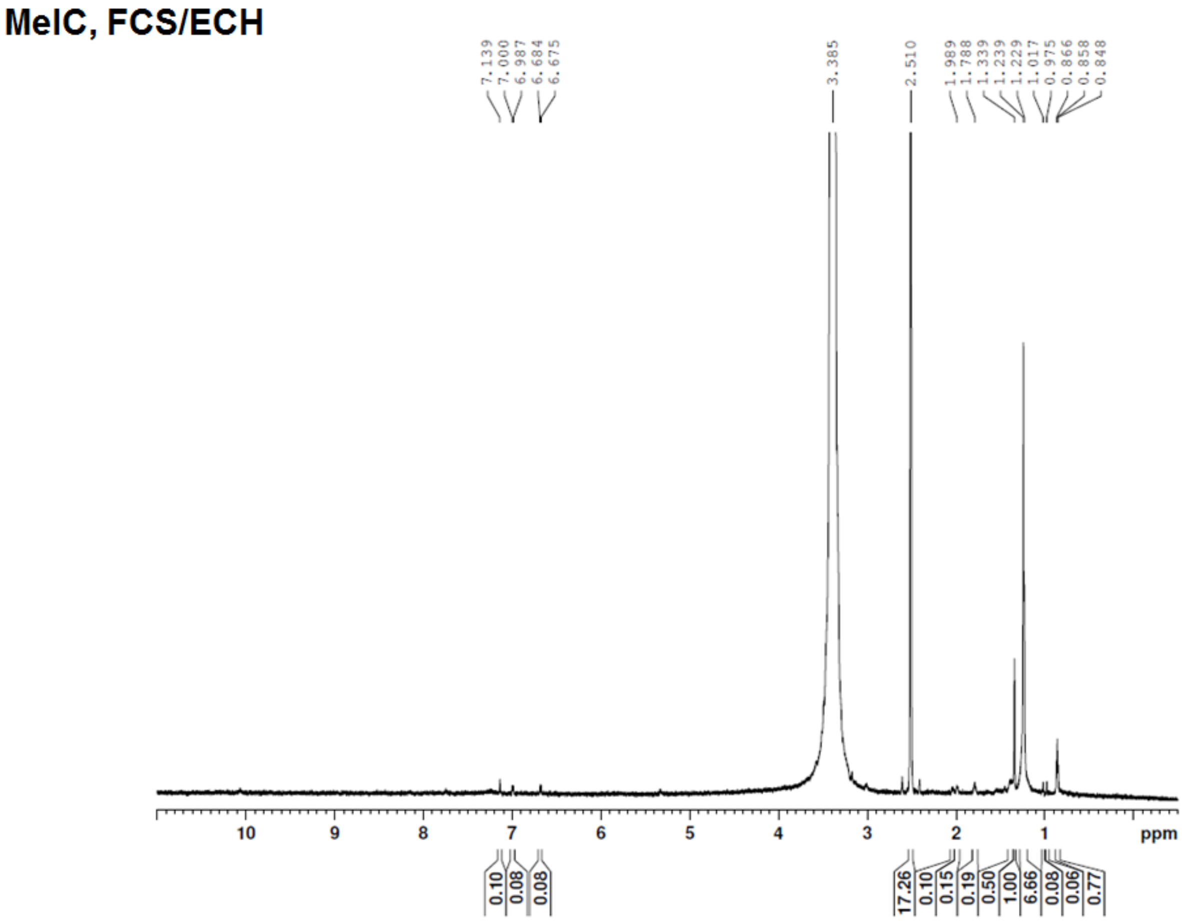
MeIC, FCS/ECH
7.139
7.000
6.987
6.684
6.675
3.385
2.510
1.989
1.788
1.339
1.239
1.229
1.017
0.975
0.866
0.858
0.848
10
9
8
7
6
5
4
3
2
1
ppm
0.10
0.08
0.08
17.26
0.10
0.15
0.19
0.50
1.00
6.66
0.08
0.06
0.77

**(C)**

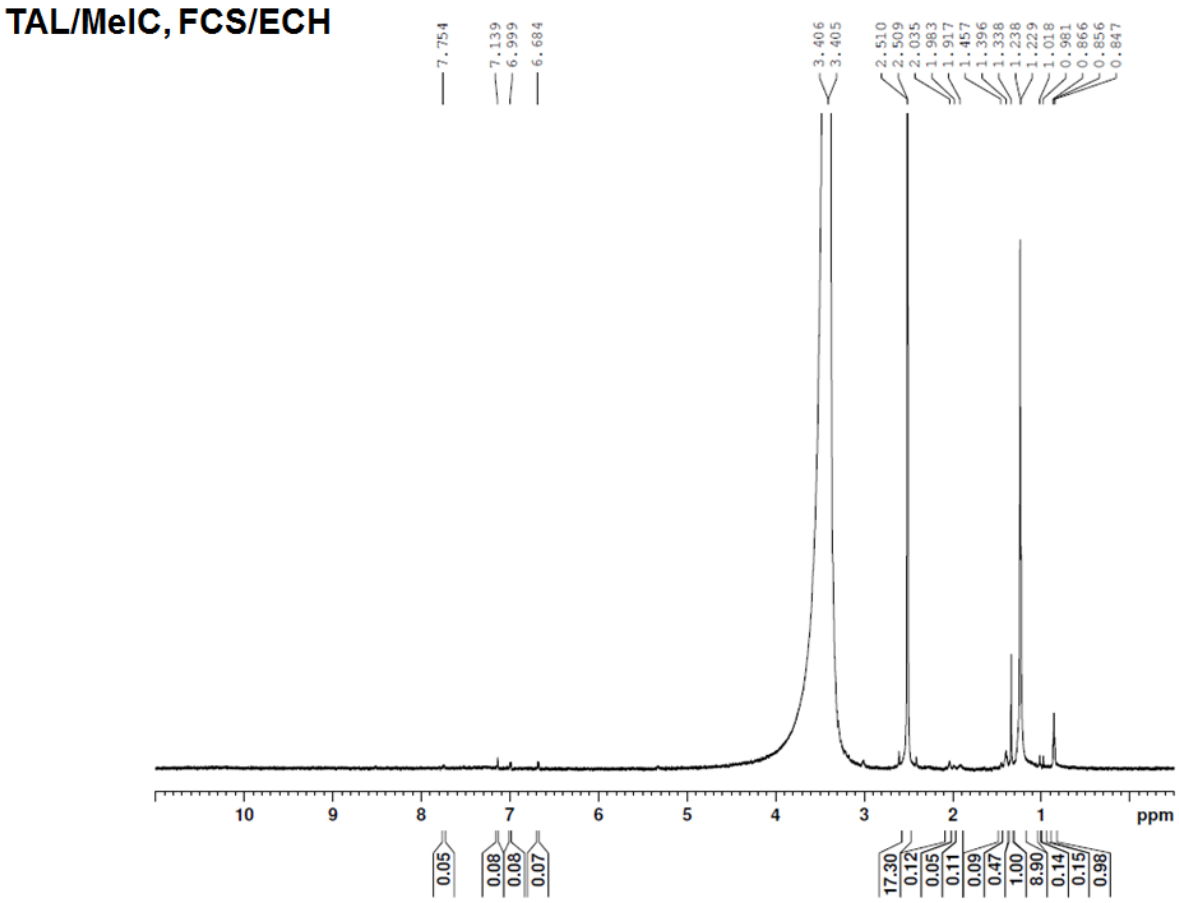
TAL/MeIC, FCS/ECH
7.754
7.139
6.999
6.684
3.406
3.405
2.510
2.509
2.035
1.983
1.917
1.457
1.396
1.338
1.238
1.229
1.018
0.981
0.866
0.856
0.847
10
9
8
7
6
5
4
3
2
1
ppm
0.05
0.08
0.08
0.07
17.30
0.12
0.05
0.11
0.09
0.47
1.00
8.90
0.14
0.15
0.98
